# Everybody's Page

**Published:** 1888-02-25

**Authors:** 


### Everybody's Page.

The division of labour, which is regarded as of such advantage in assisting our modern methods of work, may tend to make men more susceptible to nervous affections, for, by narrowing their lines and lessening their interests, it naturally makes them one-sided, and therefore imperfect.

Chronic indigestion and constipation are among the minor ills of life, and not seldom the former is the result of the latter. Anyone wanting a simple remedy without resorting to a process of physicking may try Dahl's dyspepsia cakes. They are not very palatable when eaten dry, but when soaked in tea or milk may be eaten with relish. They consist of the outer covering of several kinds of grain. For constipation they are speedily effective and give no pain.

It is always pleasant to hear of employers who look upon their workmen as more than mere machines, and we record with great satisfaction that Messrs. William Mowat and Sons, leather merchants, of Stonehaven, N.B., have just had a large room fitted up at the Carron Tan Works for the use of their workers during meal hours. The room is comfortably furnished, and has a supply of newspapers and periodicals, as well as the materials for various games.

There are many persons who persist in gorging themselves with unvarying regularity, and yet manage by the aid of what are called "dinner pills" to escape retribution for a longer or shorter period. Sooner or later, however, the process comes to an end, and, after more or less of penance in one form or another, the sinner has to betake himself to a different life as regards diet. Such men seem to think that after a long course of self-indulgence the doctor can wipe away all the effect of their past errors; but it cannot be too widely known that there is no such thing as medical absolution.

At Pforzheim, in Baden, there is carried on a large manufacture of jewellery; in fact, most of what is known as Parisian jewellery is manufactured at Pforzheim. In this town there is an excellent *Gewerbe*, or technical school, where children intended for employment in the jewellery trade receive a thorough theoretical training in their work before they enter the factory. Instruction in it is practically free; the manufacturers paying about eight shillings a-year for each of the pupils they intend to employ. The processes employed in the manufacture are numerous and elaborate; but owing to the thoroughness of the training, the pupil at the *Gewerbe* becomes an apprentice, and the apprentice a skilled workman by efforts so gradual as scarcely to be felt.

Casual wards are not luxuriantly furnished in this country, but they seem to be palatial compared with the Chinese substitutes for them to be found in the Great Imperial Almshouse at Pekin. Nearly eight hundred people are congregated there, the weather at present being so intensely cold that sleeping out-of-doors is impossible. On an average about fifty people occupy the great brick platform which, in each room, supplies the place of a bed. They are expected to lie there "straight as a corpse," and when they seem to be as closely packed as herrings in a barrel, an attendant comes along, and with his heavy feet presses two apart till a third can be wedged between. Ventilation there is none; in many of the rooms no air comes in except when someone enters or leaves the apartment. So oppressive is the air that men and boys by the score prefer the risk of freezing to death in the rooms which have neither brick platforms for beds nor paper pasted over the empty window-frames. As usual, there is "nobody to blame" for this terrible state of affairs. Looking after the destitute is everybody's business, so—nobody does it.

Some transparent soaps are very good, some very bad. Good transparent soap is prepared from ordinary soap of good quality, which is cut into shavings, dried to a large extent, and then treated with alcohol, which is afterwards evaporated, and by the process of evaporation the transparent soap is left. There are two advantages in this treatment. The first is that when the soap is cut into shavings, the soda, if any has been left, tends to get mild by contact with the air; and, further, by treating the material with alcohol, all extra soda gets out of the mixture. The substance is thus improved, and the result is a fine quality of soap, suitable for the most delicate skin. But many transparent soaps are not good. Many of those in the market are prepared hastily from oils and fats and soda, and the transparency is imparted by the addition of sugar, sometimes as much as 25 per cent., and a little petroleum.

There was a time when phrenology claimed to be a part of medical science, and enthusiastic exponents of it had hopes of seeing it placed as at least an optional class in the educational curriculum. Those dreams have vanished, and phrenology, like chiromancy and thought-reading, has become little more than a drawing-room amusement. Nevertheless, there still exists a British Phrenological Association, to whose members Mr. James Webb lately read a paper on "The Phrenology of the Poets." Mr. Webb's idea is that the cerebral organisation and characteristics of poets are complementary, and he endeavours to picture what the cranial development of the poets must have been from their writings. When you have your skull, so to speak, in one hand, and your poem in the other, it is easy to make them fit and explain the one by the other; but prophesying after the event, though safe, is rather uninteresting; and the true test of Mr. Webb's method would be for him to read the "bumps" of a few young and unrecognised geniuses, and wait for twenty years or so to see if the result corresponded with the predictions. We wonder if it would? Remembering the tale told by Harriet Martineau of her head being examined by a phrenologist who did not know her, remembering, too, the very unfavourable report this gentleman gave of the intellect of one of the cleverest women of her time, we confess to being harassed with doubts.

The paradise of the oyster-eater must be the State of Maryland. The oysters seem to like it too, for there they thrive and multiply at such a rate that the ten millions of bushels annually taken from the oyster-beds of Maryland are hardly missed. They are taken from only 190 square miles of a possible fishing-ground of 2,200 square miles. The part that oysters play in the life of some parts of that State we can hardly realise. In Baltimore there are 640 men whose regular work for eight months of the year is opening oysters. Down near Chesapeake Bay oysters are used as current coin; and the editor of one newspaper complains that a large number of the subscribers pay for the journal entirely in oysters. He receives from 150 to 200 bushels of them annually, and his family grumble at the monotonous character of their food. In old days the apprentices of Scotland made their masters promise that they should not dine on salmon more than three times a-week; the proprietor of the journal alluded to should follow their example, and insist that the readers of his paper should not pay more than a third of their subscription in oysters. We in this country, who so often long vainly for the delicious mollusc, may be glad to hear that a method of transporting oysters without injury to their freshness has been discovered. It consists in simply fastening a wire tightly round the shell. This prevents the oyster from opening, and thereby admitting air, whose action is the primary factor in their decay.

